# Sensing the oxygen and temperature in the adipose tissues – who’s sensing what?

**DOI:** 10.1038/s12276-023-01113-x

**Published:** 2023-11-01

**Authors:** Gi-Sue Kang, Hye-Ju Jo, Ye-Rim Lee, Taerim Oh, Hye-Joon Park, G-One Ahn

**Affiliations:** 1 College of Veterinary Medicine, Seoul National University, 1 Gwanak-Ro, Gwanak-Gu, Seoul, 08826 Korea; 2 College of Medicine, Seoul National University, 1 Gwanak-Ro, Gwanak-Gu, Seoul, 08826 Korea

**Keywords:** Experimental models of disease, Metabolic disorders

## Abstract

Adipose tissues, composed of various cell types, including adipocytes, endothelial cells, neurons, and immune cells, are organs that are exposed to dynamic environmental challenges. During diet-induced obesity, white adipose tissues experience hypoxia due to adipocyte hypertrophy and dysfunctional vasculature. Under these conditions, cells in white adipose tissues activate hypoxia-inducible factor (HIF), a transcription factor that activates signaling pathways involved in metabolism, angiogenesis, and survival/apoptosis to adapt to such an environment. Exposure to cold or activation of the β-adrenergic receptor (through catecholamines or chemicals) leads to heat generation, mainly in brown adipose tissues through activating uncoupling protein 1 (UCP1), a proton uncoupler in the inner membrane of the mitochondria. White adipose tissues can undergo a similar process under this condition, a phenomenon known as ‘browning’ of white adipose tissues or ‘beige adipocytes’. While UCP1 expression has largely been confined to adipocytes, HIF can be expressed in many types of cells. To dissect the role of HIF in specific types of cells during diet-induced obesity, researchers have generated tissue-specific knockout (KO) mice targeting HIF pathways, and many studies have commonly revealed that intact HIF-1 signaling in adipocytes and adipose tissue macrophages exacerbates tissue inflammation and insulin resistance. In this review, we highlight some of the key findings obtained from these transgenic mice, including *Ucp1* KO mice and other models targeting the HIF pathway in adipocytes, macrophages, or endothelial cells, to decipher their roles in diet-induced obesity.

## Obesity and hypoxia: unraveling the impact of adipose tissue inflammation

Researchers delve into the structural and functional changes in adipocytes during diet-induced obesity, which can lead to tissue hypoxia and activation of HIF. They discuss the role of HIF in various cells, including adipocytes, immune cells, and endothelial cells, in diet-induced obesity. The authors find that HIF plays a crucial role in the progression of obesity and related metabolic diseases, with potential implications for future therapeutic interventions. The review also highlights the need for further research to fully understand the complex interplay between HIF and other factors involved in obesity.

## Oxygen sensing in obesity – hypoxia and hypoxia-inducible factor (HIF)

The World Health Organization defines obesity as excessive fat accumulation that might impair health^1^. Obesity substantially increases the risk of metabolic diseases, cardiovascular diseases, musculoskeletal diseases, Alzheimer’s disease, depression, and some types of cancers^1^. Recently, the World Obesity Federation has declared obesity a chronic progressive disease that requires intervention^2^.

In the process of diet-induced obesity, adipocytes undergo major structural and functional changes, including hypertrophy, which leads to adipose tissue expansion, resulting in inefficient blood flow to the tissue^3^. This may then create tissue hypoxia, in which the affected cells in adipose tissues, such as adipocytes, immune cells, and endothelial cells, activate the transcription factor hypoxia-inducible factor (HIF) to adapt to such hypoxic conditions^4^. Indeed, visceral adipose tissues obtained from obese human individuals have demonstrated a significantly lower level of oxygen tension, accompanied by high expression of *HIF1A* and other genes involved in inflammation and fibrosis^5^. Interestingly, severe adipose tissue hypoxia detected by pimonidazole, a nitroimidazole compound that is reduced, thereby binding to sulfhydryl groups of various molecules forming pimonidazole adducts^6^, has been detected as early as 3 days in mice fed a high-fat diet^7^.

**Fig. 1:**
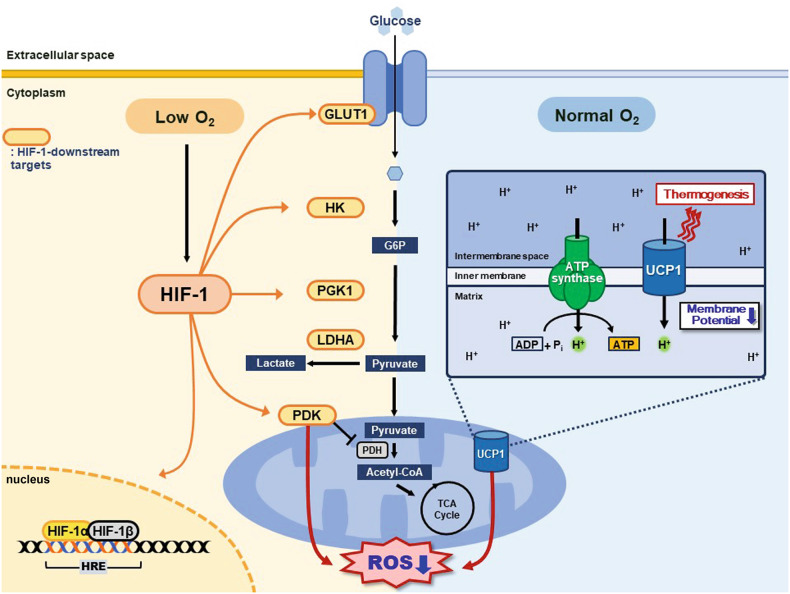
Diagram demonstrating how HIF-1 and UCP1 in a mammalian cell can regulate heat generation through mitochondrial metabolism. HIF-1 can be activated selectively under hypoxic conditions (yellow color; left side of the diagram), regulating many enzymes involved in the glycolysis pathway, including GLUT1, HK, and PDK. PDK in turn blocks pyruvate conversion to acetyl-CoA in the mitochondria, thereby decreasing oxidative phosphorylation (OXPHOS)-mediated ROS production. Under normoxic conditions (sky blue color; right side of the diagram), UCP1 can generate heat in the process of electron transport chain-mediated OXPHOS by uncoupling the proton gradient. The net result of UCP1 is also to reduce ROS production in the mitochondria.

HIF is a heterodimeric transcription factor composed of an O_2_-sensitive α subunit and an O_2_-insensitive β subunit^8^. To date, three α forms (HIF-1α, HIF-2α, and HIF-3α) are known to exist. These α forms (at least the human form) contain a basic helix-loop-helix (bHLH) domain, two Per-Arnt-Sim (PAS) domains, a PAS-associated COOH-terminal (PAC) domain, and an oxygen-dependent degradation (ODD) domain containing an NH2-terminal transactivation domain (N-TAD)^9^. Only HIF-1α and HIF-2α have a COOH-terminal transactivation domain (C-TAD)^9^. HIF-3α has been reported to exist as multiple variants, and some of the variants may lack one or more of the domains described above^9^. HIF-1α, HIF-2α, and HIF-3α protein stability is regulated by prolyl hydroxylases (PHD; Fig. 1), which require O_2_ and 2-oxoglutarate as substrates and ascorbate as a cofactor^10^. Hydroxylated Pro402 and Pro564 of the HIF-α form then allow von Hippel‒Lindau (VHL) tumor suppressor protein to bind and mediate the ubiquitination of HIF-α protein^10^ (Fig. 1). The transactivation capacity of HIF-1α and HIF-2α (but not HIF-3α because it lacks a C-TAD domain) can be further controlled by an asparagine hydroxylase (factor inhibiting HIF (FIH); Fig. 1), which also requires O_2_ and oxoglutarate as substrates, hydroxylating Asn803 in the C-TAD domain^10^. This hydroxylation at Asn803 of HIF-1α or HIF-2α prevents HIF from recruiting the transcriptional coactivators CBP and p300^10^.

Once the HIF-α form is stabilized in the cytoplasm, it binds to HIF-1β, which translocates the HIF heterodimer into the nucleus, where it binds to the hypoxia-responsive element (HRE) of the downstream target genes^10^ (Fig. 1). Although HIF-1 and HIF-2 share a number of common downstream target genes, including *vascular endothelial growth factor* (*VEGF*) and *adrenomedullin* (*ADM*)^11^, there are genes that are exclusively regulated either by HIF-1 or HIF-2. HIF-1 has been reported to regulate many genes involved in glycolysis metabolism, including *glucose transporter-1* (*GLUT-1*)*, phosphoglycerate kinase-1* (*PGK-1*), and *lactate dehydrogenase A* (*LDHA*) (Fig. 1), while HIF-2-specific downstream target genes include *erythropoietin* (*EPO*)^12^ and *adipose differentiation-related protein* (*ADRP*)^11^. HIF-3 is quite different from HIF-1 and HIF-2^13^ such that HIF-3 may compete with HIF-1 and HIF-2, thereby negatively regulating target genes of HIF-1 and HIF-2 under hypoxic conditions^13^. However, a recent study reported contradictory findings that HIF-3 can share some target genes with HIF-1 and HIF-2, such as *GLUT1*, *EPO* and *angiopoietin like-4* (*ANGPTL4*)^14^. HIF-3 does not seem to turn on HRE-driven reporter expression, indicating that HIF-3 regulates its target genes by binding non-canonical sites of cis-elements^15^.

## HIF and adipocytes

Given the importance of tissue hypoxia in adipose tissues during diet-induced obesity, a number of researchers have investigated the role of HIF in adipocytes by generating tissue-specific knockout (KO) mice targeting HIF pathways (Table 1). A number of studies have consistently demonstrated that HIF-1α expression in white adipocytes exacerbates insulin resistance and tissue inflammation (Table 1). For example, high-fat diet-fed adipocyte-specific *Hif-1α* KO mice with the aP2 promoter have demonstrated improved insulin sensitivity resistance^7,16–18^ (Table 1), increased oxidative metabolism mediated by PPAR-gamma coactivator 1 alpha (PGC-1α)^19^, and decreased tissue inflammation^7,20^. However, there are also some studies reporting contradictory findings where mice expressing the dominant negative form of human *HIF-1A* in adipose tissues exhibit increased body weight gain, insulin resistance, and adipose tissue fibrosis^20,21^.

**Table 1:** Some of the key studies published previously that investigated the adipocyte-specific disruption of HIF signaling in mice fed a high-fat diet.

Gene	Promoter	Methods	Effects	Phenotype	References
*Hif-1α*	aP2	Cre-ERT2/loxP	↓ HIF-1α in adipocytes upon tamoxifen treatment	↓ body weight gain ↑ PGC-1α protein expression and mitochondrial functions	^19^
		Cre/loxP	↓ HIF-1α in adipocytes	↑ insulin sensitivity ↓ adipose tissue inflammation	^7,17^
		knock-in of human *HIF1A* lacking the DNA binding domain	↓ HIF-1α in adipocytes	↑ body weight gain ↓ insulin sensitivity ↓ core body temperature ↓ Pgc-1α and UCP1 gene and protein expression ↑ adipose tissue fibrosis	^20,21^
*Hif-1β*	aP2	Cre/loxP	↓ HIF-1α in adipocytes (theoretically ↓HIF-1α, HIF-2α, and HIF-3α in adipocytes)	↓ body weight gain ↑ insulin sensitivity	^16,18^
*Hif-2α*	Fabp4	Cre/loxP	↓ HIF-2α in adipocytes	↑ body weight gain ↓ insulin sensitivity ↑ adipose tissue inflammation and hypoxia ↓ adipose tissue vascularization	^22^
	N/A	knock-in amino acid substituted (S304M) human HIF-2α	↓ HIF-2 activity in all tissues	↑ body weight gain ↑ adipogenesis	^23^
*Phd2*	aP2	Cre/loxP	↑ HIF-1α and HIF-2α in adipocytes	↓ body weight gain ↑ insulin sensitivity ↑ vascularity in adipose tissues ↑ glycolytic gene expression ↑ oxygen consumption	^24^
*Vhl*	aP2/Fabp4	Cre/loxP	↑ HIF-1α, HIF-2α, and HIF-3α in adipocytes	No difference in the body weight gain compared to WT mice ↓ visceral fat mass (but not brown adipose tissues) Cardiomegaly (HIF-2α dependent) ↓ blood glucose levels (more pronounced in female mice) ↓ serum glycerol levels ↑ circulating lymphocytes (more pronounced in female mice)↑ inflammatory gene expression in adipose tissues	^25^

*N/A* not applicable.

While there are fewer studies on the role of HIF-2α in adipocytes, the results seem consistent: intact HIF-2 signaling in adipocytes has a protective role against diet-induced obesity (Table 1). For example, adipocyte-specific *Hif-2α* KO mice with the fatty acid binding protein 4 (Fabp4) promoter exhibit increased body weight gain, insulin resistance, and tissue inflammation under high-fat diet conditions^22^ (Table 1). Another study that used knock-in of a single amino acid substitution (S305M) within the *HIF-2α* PAS-B domain in all tissues in mice reported that these mice gain more body weight^23^ (Table 1) and exhibited increased expression of genes involved in adipogenesis, including *adiponectin*, *leptin*, *lipase*, *Fabp4, peroxisome proliferator-activated receptor gamma (Pparγ), CCAAT/enhancer-binding protein alpha (Cebpα), CCAAT/enhancer-binding protein beta (Cebpβ)*, and *ATP citrate lyase (Acly)*, in visceral adipose tissues^23^.

Mice with deletion of *Phd2*, thereby constitutively upregulating HIF-1α, HIF-2α, and HIF-3α in adipocytes, exhibited decreased body weight gain, improved insulin sensitivity, and increased oxygen consumption (but not carbon dioxide production) upon high-fat diet feeding^24^ (Table 1). Visceral adipose tissues isolated from these mice exhibited increased vascularity, decreased adipose tissue macrophage (ATM) infiltration, and increased expression of many glycolytic genes, including *Glut-1*, *Pdk-1*, *Gapdh*, and *Ldha*^24^.

Adipocyte-specific *Vhl* KO mice using aP2 and Fabp4 dual promoters, whereby HIF-1α, HIF-2α, and HIF-3α are constitutively upregulated in the affected adipocytes, demonstrate no reduction in body weight gain, although visceral fat mass is significantly decreased^25^ (Table 1). These mice have a profound cardiac hypertrophy phenotype^25^, which may have compromised the investigators’ attempt to clearly dissect the role of HIF in adipocytes.

## HIF and stromal cells in adipose tissues

### Adipose tissue macrophages (ATM)

ATM are known to be the most abundant immune cells in the adipose tissues of obese individuals^26^. While they comprise fewer than 10% of the total immune cells in lean adipose tissues, this fraction rises to more than 50% in obese adipose tissues^26^. Furthermore, the number of ATM has been shown to correlate well with total adiposity and adipose cell size^27^. Macrophages are recruited to adipose tissues by various cytokines and chemokines, including C-X-C motif chemokine 12 (CXCL12)^28^ and semaphorin 3E^29^ secreted from adipocytes, which may be activated by the HIF transcriptional program. Generally, macrophages are known to be polarized either to the proinflammatory M1 or anti-inflammatory M2 phenotype^30^. In diet-induced obesity, extensive evidence suggests that M1-polarized macrophages dominate the adipose tissues of obese mice^27^ and humans^31^. A recent study with single-cell RNA sequencing technology, however, demonstrated that several classical M1/M2 signature genes are rarely expressed in ATM, although inflammatory signaling pathways, including nuclear factor kappa-light-chain-enhancer of activated B cells (NF-κB), Rho GTPases, mammalian target of rapamycin (mTOR), and p38 mitogen activated protein kinase (MAPK) signaling pathways, do seem to be ‘activated in M1-like’ and ‘transitional M1-like’ ATM in obese mice but not in lean mice^32^. It has also been suggested that a high-fat diet leads to an endoplasmic reticulum stress response in adipocytes, which may in turn affect macrophage polarization^33^. ATM often exhibit a crown-like structure, which has been shown to be a result of scavenging lipid droplets released from dead adipocytes, forming multinucleated giant cells^34^.

As with adipocytes, a number of researchers have generated macrophage-specific *Hif*-KO mice using the lysozyme M (LysM) promoter to study the role of HIF in ATM in obesity (Table 2). Takikawa and colleagues^35^ demonstrated that HIF-1 deficiency in ATM leads to improved whole-body glucose clearance and tissue insulin sensitivity, reduced tissue inflammatory gene expression, enhanced angiogenesis and reduced hypoxia in adipose tissues (Table 2). Sharma and colleagues^36^ observed less ATM infiltration and Ki67-positive proliferating cells in the white adipose tissues (WAT) of macrophage-specific *Hif-1α* KO mice (Table 2). In contrast to these studies, Kihira and colleagues^17^ have reported that glucose clearance and ATM infiltration signatures are not different between the mutant (macrophage-specific *Hif-1α* KO mice) and their wild-type (WT) counterpart mice (Table 2). Nonetheless, all studies have commonly found that there is no difference in body weight gain between mutant and WT mice upon high-fat diet feeding^17,35^. The role of HIF-2α in macrophages in obesity is somewhat unclear. High-fat diet-fed macrophage-specific *Hif-2α* KO mice with the LysM promoter demonstrated no difference in insulin sensitivity between the mutant and WT mice^22^, whereas *Hif-2α*^+/−^ heterozygous mice treated with clodronate liposomes, a macrophage-depleting agent, demonstrated improved glucose clearance^37^ (Table 2). Macrophage-specific *Phd2* KO mice fed a high-fat diet have been reported to exhibit increased insulin resistance, adipose tissue fibrosis, and tissue inflammation^38^ (Table 2).

**Table 2:** Some of the important studies previously published that highlight the role of HIF in stromal cells in diet-induced obesity.

Target cells	Gene	Promoter/Methods	Effects	Phenotype	Body weight gain upon high-fat diet feeding	References
ATM	*Hif-1α*	LysM	↓ HIF-1α in macrophages	↑ insulin sensitivity ↑ vascularity in the adipose tissues ↓ tissue hypoxia	No effect	^35^
				↓ ATM infiltration ↓ Ki67-positive proliferating cells	No effect	^36^
				− glucose clearance − ATM signature	No effect	^22^
	*Hif-2α*	LysM	↓ HIF-2α in macrophages	− insulin sensitivity − vascularity in the adipose tissues	No effect	^37^
		*Hif-2α*^+/−^ mice treated with clodronate liposomes	Depletion of HIF-2α-positivity in macrophages	↓ ATM infiltration ↑ insulin sensitivity	No effect	^38^
	*Phd2*	LysM	↑ HIF-1α, HIF-2α, HIF-3α	↓ insulin sensitivity	No effect	^36^
Endothelial cells	*Hif-2α*	Stem cell leukemia	↓ HIF-2α in endothelial cells	− insulin sensitivity − changes in endothelial cell numbers	No effect	^22^

-, no changes, clodronate liposome, a macrophage-depleting agent.

### T cells

T cells are the second largest population of immune cells in obese adipose tissues^26^. Obesity has been shown to dramatically increase the frequency of T helper (Th) 1, Th17, and CD8 + T cells in WAT, whereas the frequency of Th2 and regulatory T (Treg) cells is decreased in WAT^39^. As T-cell activation requires aerobic glycolysis and glutamine catabolism triggered by downstream signaling cascades of the T-cell receptor, costimulatory molecules and cytokines^40^, HIF-1 has been shown to be essential in promoting glucose uptake and glycolysis to mediate the cytotoxic T-cell response^41^. Furthermore, HIF-1α is strongly induced in T cells undergoing Th17 differentiation, while the lowest levels are detected in cells undergoing Treg differentiation^42^. Despite the importance of HIF in the regulation of T-cell activation and inflammation^43^, there are no reports to date, as far as the authors know, investigating how HIF in T cells impacts diet-induced obesity.

### Endothelial cells

Evidence suggests that obesity is accompanied by endothelial cell dysfunction and decreased vascular density^44^ and that modulation of endothelial cell function through some of the key molecules, including transcription factors (PGC-1α, PPARγ, and NF-κB), angiogenic signaling (VEGF/vascular endothelial growth factor receptor 2 (VEGFR2) and angiopoietin 2 (ANGPT2)/TEK tyrosine kinase (TIE2)), insulin signaling (insulin receptor), and mediators of fatty acid transporters (CD36 and PPARγ), is sufficient to regulate the progression of obesity^45^.

Endothelial-specific *Hif-1α* KO mice with the Tie2 promoter have demonstrated elevated basal glucose levels, decreased glucose clearance, delayed insulin release in the blood upon glucose load, and decreased glucose uptake into tissues, including the brain and heart^46^ (Table 2). Endothelial-specific *Hif-2*a KO mice with a stem cell leukemia promoter, on the other hand, exhibited no differences in body weight gain, fat mass, insulin sensitivity, or tissue vascularity compared to their WT controls when fed a high-fat diet^22^ (Table 2).

## Factors other than molecular oxygen that activate HIF in obesity

HIF is well known to be regulated by the level of molecular oxygen. However, there are several factors other than molecular oxygen that can activate HIF. Some of these factors include reactive oxygen species (ROS) reducing Fe^2+^ to Fe^3+^, thereby inhibiting PHD activity^47^, increased concentrations of succinate and fumarate inhibiting the PHD reaction^22^, lipopolysaccharide^48^ activating the protein kinase C and phosphatidylinositol 3-kinase pathway, thereby increasing HIF-1α transcription and translation^49^, sphingosine-1-phosphate from apoptotic cell debris activating nuclear factor of activated T cells and hence HIF-1α translation^50^, and proinflammatory cytokines such as tumor necrosis factor-α and interleukin-1β increasing DNA binding of HIF-1^51,52^.

From the obesity perspective, He and colleagues^53^ have demonstrated that insulin treatment alone is sufficient to increase HIF-1α mRNA and protein levels in differentiated 3T3-L1 adipocytes^53^. Interestingly, high glucose levels in normal cultured cells have been reported to reduce HIF-1α protein levels and increase mitochondrial ROS production^54^, perhaps through elevated fatty acid metabolism, lowering succinate availability for HIF-1α protein stabilization^55^. In addition, an increase in 2-methylglyoxal that accompanies high glucose conditions such as obesity and diabetes stimulates HIF-α degradation by inhibiting HIF-α-HIF-1β dimer formation and recruitment of the p300/CBP regulatory complex^56^.

## HIF inhibitors as a potential anti-obesity drug

Given the importance of HIF in obesity, some HIF inhibitors have been shown to be effective in attenuating the obese phenotype in mice fed a high-fat diet. PX-478, a selective inhibitor of HIF-1α, effectively reduced weight gain accompanied by decreased fibrosis and inflammation^57^. YC1, a suppressor of HIF-1α accumulation^58^, has also been shown to induce lipolysis in Raw264.7 foam cells^58^ and rat visceral fat cells^59^. PT2399, an HIF-2α-specific antagonist, has been shown to protect mice from diet-induced obesity by decreasing body weight, adipogenesis and lipogenesis when administered for 8 weeks^23^. Digoxin, a cardiac glycoside that increases heart contraction forces by reversibly inhibiting the activity of the sodium-potassium ATPase pump, also identified through compound library screening as a small molecule HIF-1 inhibitor^60^, has been reported to prevent diet-induced obesity in mice, although the suggested mechanism is through inhibition of the IL-17A phosphorylation of PPARγ in adipocytes^61^. Berberine, an ingredient in the Chinese herbal medicine *Coptis chinensis*^62^, and anti-sense oligonucleotides to HIF-1α^63^ have been shown to attenuate body weight gain in mice fed a high-fat diet through inhibition of HIF-1α expression.

## Temperature sensing in obesity – uncoupling protein (UCP) in adipose tissues

Uncoupling proteins (UCP) are members of the mitochondrial anion carrier family^64^, and five UCP homologs have been identified to date. UCP1, the first UCP identified and the most extensively studied member, is predominantly expressed in brown adipose tissue (BAT) and allows an alternative route for protons to enter other than ATP synthase, generating heat as a result of the dissipation of the energy from the electrochemical gradient^65^ (Fig. 1). In doing so, free fatty acids have been shown to act as secondary messengers to facilitate proton conductance mediated by UCP1, whereas the binding of purine nucleotides such as ADP and GDP has been shown to inhibit UCP1 activity^65,66^. UCP1 is regulated at the transcriptional level, and many physiological signals, including the cold-induced release of catecholamines (epinephrine and norepinephrine), act on β3-adrenergic receptors in BAT^66^. Recent evidence suggests that UCP1 can also be expressed in WAT under certain circumstances, such as cold-induced catecholamine activation of β3-adrenergic receptors, exercise-induced release of fibroblast growth factor (FGF)−21 acting on FGF receptors, and thyroid hormone synergizing with the activation of β3-adrenergic receptors^67^. Under these conditions, the phenomenon is also known as ‘browning’ of WAT or ‘beige adipocytes’; white adipocytes undergo lipolysis and FFA release through induction of UCP1 expression^67^. *Ucp1* KO mice have been shown to be non-obese upon high-fat diet challenge and extremely cold sensitive^68^. UCP2 expression has been observed in many tissues, including adipose tissue, muscle, heart, kidney, digestive tract, brain, spleen, and thymus^69^, while UCP3 is primarily expressed in skeletal muscle in humans and rodents and in the heart and BAT in rodents^70^. Although UCP2 and UCP3 have 59% and 57% amino acid identity, respectively, to UCP1^71^ – hence, they were originally thought to be thermogenic genes – it has been revealed that they are unlikely to be involved in adaptive thermogenesis based on the following evidence: the mitochondrial uncoupling by UCP2 has been shown to occur at much higher expression levels than the endogenous one^66^ and *Ucp2*^72^ or *Ucp3*^73^ KO mice are not cold sensitive.

## Relationship between oxygen and temperature sensing in adipocytes – a possible link between HIF and UCP

Can adipocytes sense oxygen levels and temperature at the same time, and if so, what would be the consequences? It seems that molecular oxygen, glucose, and fatty acids are commonly shared signaling molecules by both HIF and UCP. Thermogenically activated BAT has been shown to take up large amounts of glucose, although there is no significant release of lactate^74^, indicating that glucose is utilized mainly in oxidative but not glycolytic metabolism. Then, is UCP required for glucose uptake by adipocytes? Inokuma and colleagues^75^ demonstrated that *Ucp1* KO mice lack the stimulatory effect of norepinephrine on glucose uptake into BAT, suggesting that UCP1 is required for glucose uptake into adipocytes. Glucose taken up by adipocytes may then be utilized to synthesize tricarboxylic acid intermediates or converted to free fatty acids^76^. Since HIF-1 regulates the expression of GLUT-1, a transporter essential for glucose uptake^11^, and pyruvate dehydrogenase (PDH), an enzyme that converts pyruvate to acetyl-CoA^77^, HIF-1 may promote UCP1-mediated glucose uptake and oxidative phosphorylation (Fig. 1). However, given that HIF-1 activation occurs with either low oxygen tension or other factors, such as ROS, it is unlikely that HIF-1 and UCP1 cooperate in adipocytes at the same time. In line with this, the fact that free fatty acids, which are required for UCP1-mediated thermogenesis, prevent hypoxia-induced HIF-1α accumulation^55^ indicates a mutually exclusive relationship between HIF-1 and UCP1. Interestingly, however, there are a couple of studies demonstrating a possible interlinked regulation between UCP and HIF. For example, Han and colleagues^78^ have reported that cold exposure or CL-316,243, a β3-adrenoceptor agonist, treatment in mice results in an increased protein expression of HIF-1α and HIF-2α in thermogenic adipose tissues (inguinal and brown adipose tissues, but not in visceral adipose tissues), which closely coincided with the increased expression of UCP1 in these tissues. Similarly, Basse and colleagues^79^ demonstrated that isoproterenol, a β-adrenergic stimulator that normally increases UCP1 expression in adipocytes^80^, not only increases HIF-1α mRNA and protein levels but also increases the expression of glycolytic genes, including *Glut-1*, *hexokinase* (*Hk*), *Pgk-1*, and *Ldha*. In another study, leptin-deficient *ob/ob* mice treated with deferoxamine, a well-known iron chelator and PHD inhibitor, which thereby stabilizes HIF-α^81^, demonstrated increased UCP1 protein expression and mitochondrial biogenesis and improved insulin sensitivity markers in adipose tissues^82^.

Nonetheless, the end result of the signaling by both HIF-1 and UCP seems to be a reduction in mitochondrial ROS production (Fig. 1). UCP has been generally reported to decrease mitochondrial ROS by inducing proton leakage (Fig. 1), which allows mitochondria to avoid the oversupply of electrons/reducing equivalents into respiratory complexes and minimizes the likelihood of electron interaction with oxygen^83^. UCP2 and UCP3 have also been reported to catalyze proton leakage, thereby decreasing mitochondrial ROS^84^. HIF-1 can decrease mitochondrial ROS production by inhibiting the uptake of pyruvate into the mitochondria through the expression of pyruvate dehydrogenase kinase-1 (PDK1), which inactivates PDH, thereby inhibiting the conversion of pyruvate to acetyl-CoA in the tricarboxylic acid cycle^77^ (Fig. 1).

## Conclusion

Based on the critical role played by HIF and UCP in physiological as well as pathological settings, there are numerous studies on how each of these molecules is regulated under the setting of diet-induced obesity, for example, by treatment with free fatty acids. However, one should bear in mind that the role of these molecules in obesity can be significantly different from those in, for example, cancer. For instance, while fatty acid metabolism decreases HIF-1α protein stabilization in the setting of obesity^55^, treatment of hepatocellular carcinoma cells with free fatty acids, such as oleic acid, can increase HIF-1α protein expression under both normoxia and hypoxia^85^. One solution to this complex situation may be to use an appropriate reporter system. With the latest technology, mice expressing ubiquitous ROSA26 ODD-driven luciferase^+/−^ have been created, and these mice emit bioluminescence signals in mice breathing 8% oxygen or injected with hypoxia mimetics^86^. ThermoMouse^87^ and a dual *Ucp1* reporter mouse^88^ expressing *Ucp1*-driven luciferase 2 and *Ucp1*-driven firefly luciferase and near-infrared red fluorescent protein, respectively, have been generated; these mice demonstrate the highest luciferase activity and thereby UCP1 expression in BAT of mice exposed to cold temperature, CL-316,243, or rosiglitazone (a PPARγ agonist). Using these transgenic mice, the development of HIF inhibitors or UCP1 activators as anti-obesity drugs may be facilitated, although low sensitivity due to the penetration depth in these mice remains a major obstacle to overcome. There may be a growing interest in developing UCP1 activators as a therapeutic strategy to combat diabetes and obesity. In fact, dinitrophenol (DNP), a benzene-based chemical, was widely used as a weight-loss drug in the 1930s^89^. It turned out that it acts as a proton permeabilizer, thereby uncoupling oxidative phosphorylation and causing fat calories to be dissipated as heat^89^. However, DNP is associated with some serious side effects, including rare idiosyncrasies and loss of vision^90^. With the known mechanism of UCP and thermogenesis, Cavalieri and colleagues^91^ utilized protein thermostability shift analysis to identify novel activating ligands of UCP, including tetradecylthioacetic acid (a β-oxidation-resistant synthetic fatty acid that activates PPAR), TUG-891 (a G protein-coupled receptor 120 agonist), and ibuprofen (a widely used non-steroidal anti-inflammatory drug). Retinoic acid^92^ and the cJun kinase inhibitor SP600125^93^ have also been reported as UCP1 activators. Although further studies of these compounds are warranted, it would be exciting if these compounds reveal paths to treat and prevent diet-induced obesity.
